# Spoligotype database of Mycobacterium tuberculosis: biogeographic distribution of shared types and epidemiologic and phylogenetic perspectives.

**DOI:** 10.3201/eid0703.010304

**Published:** 2001

**Authors:** C Sola, I Filliol, M C Gutierrez, I Mokrousov, V Vincent, N Rastogi

**Affiliations:** Institut Pasteur de Guadeloupe, Pointe poundà Pitre, Guadeloupe. csola@pasteur.gp

## Abstract

We give an update on the worldwide spoligotype database, which now contains 3,319 spoligotype patterns of Mycobacterium tuberculosis in 47 countries, with 259 shared types, i.e., identical spoligotypes shared by two or more patient isolates. The 259 shared types contained a total of 2,779 (84%) of all the isolates. Seven major genetic groups represented 37% of all clustered isolates. Two types (119 and 137) were found almost exclusively in the USA and accounted for 9% of clustered isolates. The remaining 1,517 isolates were scattered into 252 different spoligotypes. This database constitutes a tool for pattern comparison of M. tuberculosis clinical isolates for global epidemiologic studies and phylogenetic purposes.


### Spoligotype Database of *Mycobacterium tuberculosis*: Biogeographic Distribution of Shared Types and Epidemiologic and Phylogenetic Perspectives

**Christophe Sola,* Ingrid Filliol,* Maria Cristina Gutierrez,† Igor Mokrousov,* Véronique Vincent,† and Nalin Rastogi***
*Institut Pasteur de Guadeloupe, Pointe à Pitre, Guadeloupe; and †Centre National de Référence des Mycobactéries, Institut Pasteur, Paris, France

We give an update on the worldwide spoligotype database, which now contains 3,319 spoligotype patterns of *Mycobacterium tuberculosis* in 47 countries, with 259 shared types, i.e., identical spoligotypes shared by two or more patient isolates. The 259 shared types contained a total of 2,779 (84%) of all the isolates. Seven major genetic groups represented 37% of all clustered isolates. Two types (119 and 137) were found almost exclusively in the USA and accounted for 9% of clustered isolates. The remaining 1,517 isolates were scattered into 252 different spoligotypes. This database constitutes a tool for pattern comparison of *M. tuberculosis* clinical isolates for global epidemiologic studies and phylogenetic purposes.

In 1997, 8 million new cases of tuberculosis (TB) were reported worldwide; 3.5 million cases were considered highly contagious (1). With Africa and some countries having up to 20% of their populations infected with HIV, AIDS will have a major impact on TB in coming years (2). Emergence of multidrug-resistant (MDR) strains of *Mycobacterium tuberculosis* is also of great epidemiologic concern (3). In this context, molecular fingerprinting of *M. tuberculosis* complex isolates is a powerful tool that permits detection of transcontinental spread of TB (4) and outbreaks (5). Our laboratory has described a preliminary spoligotyping database that suggested the biogeographic specificity of some of the spoligotypes from the Caribbean (6). The initial aim of this work was twofold. First, such an inventory was mandatory to detect and estimate the relative importance of TB of foreign origin in the French Caribbean. Although the incidence of TB in Martinique and Guadeloupe is comparable with that in metropolitan France (approximately 10/100,000 new cases each year), this region is part of an area of Latin America and the Caribbean with high TB prevalence. Second, we used spoligotyping results to infer potential phylogenetic relationships of *M. tuberculosis* strains in the Caribbean region and the history of TB by using molecular markers. An updated database could also be helpful in developing new statistical approaches in the field of population genetics of circulating *M. tuberculosis* clinical isolates.

By systematically analyzing published spoligotypes, we have now collected 3,319 spoligotyping patterns of various origins in a single database, essentially from Europe and the USA (Table 1). This database includes 259 shared types containing 2 to 476 patterns (because of the size of this database, a graphic of it appears online only, at http://www.cdc.gov/ncidod/EID/vol7no3/sola_data.htm). The main database also includes 540 “orphan patterns” (clinical isolates showing a unique spoligotype), for a current total of 799 distinct spoligotype patterns. This article describes the nomenclature and phylogenetic reconstruction of these 259 shared types.

Address for correspondence: Nalin Rastogi or Christophe Sola, Unité de la Tuberculose et des Mycobactéries, Institut Pasteur de Guadeloupe, Morne Jolivière, BP 484, 97165 Pointe-à-Pitre, Cedex, Guadeloupe; fax: 590-893880; e-mail: rastogi@pasteur.gp; csola@pasteur.gp

### The Database

Spoligotyping based on the variability of the Direct Repeat (DR) locus and analysis of a variable number of tandem DNA repeats (VNTR) of *M. tuberculosis* were performed according to the original protocols (7,8). For the construction of the database, spoligotyping results were entered into Excel spreadsheet files in chronological order, according to the availability of results from published articles and our own investigations. The database was searched regularly for new shared types, i.e., identical spoligotypes shared by two or more patient isolates. For phylogenetic reconstruction, the spoligotyping results were entered into Recognizer software of the Taxotron package (Taxolab, Institut Pasteur, Paris), as recommended (9). The “1-Jaccard” Index was calculated for each pairwise comparison of patterns (10), and the neighbor-joining algorithm was used for building trees (11).

The source of the data and its representativeness are shown in Table 1. Of 3,319 individual spoligotypes in our database, most (2,418 [73%]) were either from Europe (1,142 [34%]) or the USA (1,283 [39%]). Spoligotypes shared between the USA and Europe totaled 1,286 isolates distributed among 45 shared types (Europe, n=461; USA, n=825). A statistical

Table 1. Source of data for 3,319 spoligotypes of *Mycobacterium tuberculosis* used to generate the database of 259 shared types

| No. of isolates | Origin[a] | Year | Reference |
|---|---|---|---|
| 136 | Denmark | 1999 | 31 |
| 147 | Italy | 1999 | 32 |
| 157 | Cuba | 1998 | 33 |
| 1[b] | Philippines | 1997 | 34 |
| 3 | Peru | 1998 | 35 |
| 18 | USA | Unpublished | R. Frothingham |
| 105 | France | 1997 | 36 |
| 167 | United Kingdom | 1997 | 37 |
| 296 | France | Unpublished | This study |
| 28 | Zimbabwe | 1998 | 38 |
| 32 | Guinea-Bissau | 1999 | 25 |
| 118 | The Netherlands | 1997 | 7 |
| 68 | Various countries | 1999 | 15 |
| 58 | France | Unpublished | J. Maïsetti & B. Carbonnelle |
| 62 | Russia | Unpublished | O. Narvskaya |
| 84 | West Africa | 1999 | 39 |
| 5 | Thailand | Unpublished | P. Palittapongarnpim |
| 14 | Romania | 1997 | 40 |
| 17 | Brazil | 1999 | 41 |
| 5[b] | Spain | Unpublished | S. Semper & C. Martin |
| 1,283 | USA | 2000 | 12 |
| 1[b] | United Kingdom | 1999 | 42 |
| 19 | The Netherlands | 1998 | 43 |
| 1[b] | The Netherlands | 1999 | 19 |
| 69 | Far East Asia | 1995 | 44 |
| 69 | Caribbean | 1999 | 6 |
| 356 | Caribbean | Unpublished | This study |

[a]Although a potential sampling bias cannot be excluded, the sampling of isolates and their representativeness (in order of description) was as follows: Denmark, of 249 isolates described with a low copy number of IS*6110* collected since 1992 (exhaustivity 93%), 24 shared types, representing 136 spoligotypes, were retained (9 other shared types, representing 49 isolates that were found exclusively in Denmark (S1,S2,S4,S19,S22,S23,S27,S30,S33), were not included in the present analysis; Italy, of 158 isolates from 156 patients in Verona collected during 1996-1997, 147 spoligotypes were retained; Cuba, of 160 isolates typed (obtained from a pool of 578 smear-positive sputa collected during 1994-1995), 157 spoligotypes described (exhaustivity 36%) were retained; Philippines, no data except for a single spoligotype available; Peru, of 29 strains isolated during 1995-1996 from the sputa of patients in Lima and Cuzco, only 3 were retained in this study since the remaining isolates shared spoligotypes with patients in Texas (12) and are included in the 1,283 Texan profiles; USA, 18 clinical isolates from the collection of R. Frothingham (representativeness unknown); France, 111 isolates from 105 hospitalized patients in Paris obtained during 1993 (patients were from three major hospitals that represented 5% of the total public hospital beds in Paris); United Kingdom, 167 isolates from all the culture-positive tuberculosis (TB) patients from three large hospitals in northwest London (without any indication of period of recruitment); France, 296 isolates sent for reference purposes during a 3-year period to the Centre National de Référence des Mycobactéries, Institut Pasteur, Paris; Zimbabwe, 28 spoligotypes obtained directly from sputum samples during a 1-month recruitment period (December 1995) of sputum-positive TB cases representing 20% of all cases; Guinea-Bissau, of 229 spoligotypes obtained from samples of 900 patients with suspected TB cases during 1989-1994, only 32 spoligotypes were fully described by the authors, and were retained for the analysis; the Netherlands, 118 isolates of unspecified representativeness from the collection of National Institute of Health (RIVM, Bilthoven); international multicenter study, 68 of 90 isolates from 38 countries representing the five continents; France, 58 isolates during a 1-year (1999) recruitment in the University Hospital of Angers; Russia, 62 isolates representing the St. Petersburg area collected during 1997-1999; West Africa, 84 isolates from Ivory Coast and around Dakar, Senegal, collected during 1994-1995; Thailand, 5 isolates from northern Thailand (unknown representativeness); Romania, 14 isolates of unknown representativeness; Brazil, 17 spoligotypes out of 91 isolates from a São Paulo hospital in 1995 (unknown representativeness); Spain, 5 multidrug-resistant isolates (unknown representativeness); USA, 1,429 clinical isolates from 1,283 patients during 1994-1999 that are part of an ongoing population-based study in Houston, Texas; United Kingdom, a single spoligotype from ancient DNA extracted from a bone sample; the Netherlands, 19 spoligotypes obtained from paraffin-wax embedded tissue samples previously collected during 1983-1993 (unknown representativeness); the Netherlands, a single spoligotype from a previous study (unknown representativeness); Far East Asia, 69 isolates from China and Mongolia obtained during 1992-1994 (unknown representativeness); Caribbean, 425 clinical isolates from a population-based ongoing study that includes all cultures isolated in Guadeloupe, Martinique, and French Guiana since 1994 and covers a 1 million population (exhaustivity 100%). Some isolates in this pool came from patients from other countries (essentially neighboring countries such as Haiti, Dominican Republic, Brazil, Commonwealth of Dominica, Barbados, and Surinam).

[b]Description of a given spoligotype without precise number of isolates within this type.

analysis was performed for the 1,286 isolates to evaluate the biogeographic specificity of the shared types and assess potential sampling bias by using a sample homogeneity test derived from the chi-square test (see below).

### Results and Discussion

### Description of Database

The 3,319 spoligotypes were grouped into 259 shared types containing 2,779 (84%) of the isolates and 540 (16%) orphan spoligotyping patterns (clinical isolates showing a unique spoligotype; results not shown; see online graphic of database, http://www.cdc.gov/ncidod/EID/vol7no3/sola_data.htm). This gives a current total of 799 distinct spoligotype patterns in our database.

The distribution of shared types, their respective sizes, and their relative distribution in different locations (distinct countries or geographic regions) are summarized in Figure 1. The 24 most frequent shared types totaled 1,804 (65%) isolates (Figure 1A); 7 types were highly frequent, representing 1,250 (45%) isolates. The Beijing type (type 1) was most frequent and represented 18% of isolates. Two types (119 and 137), which were almost exclusively found in the USA, accounted for 9% of isolates and may be specific for American populations or outbreaks (12). Types 53 and 50 accounted for 8% and 6% of isolates and were found in 17 and 15 locations, respectively. Two other types (types 42 and 47)

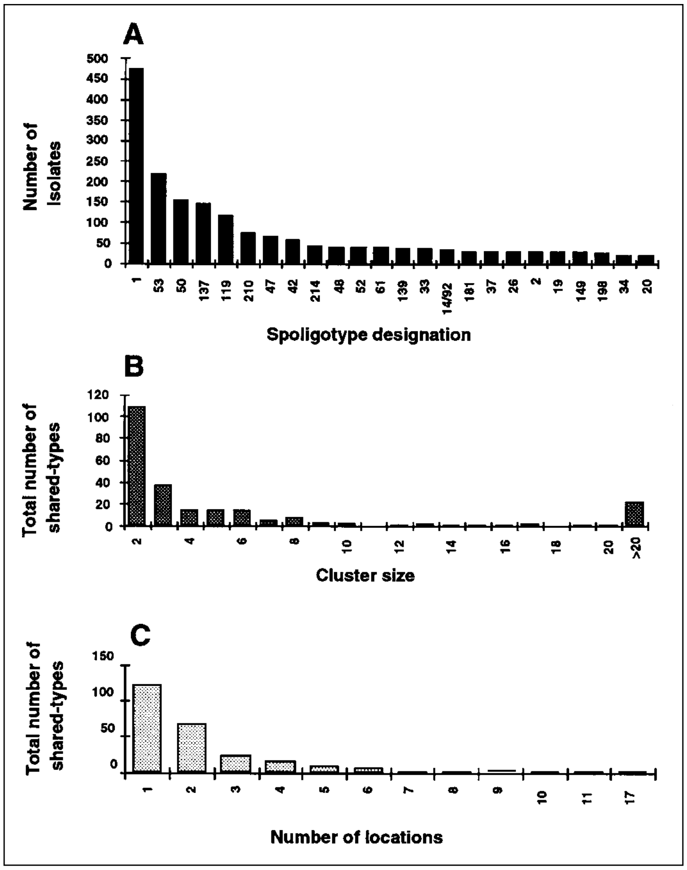

Figure 1. Histograms derived from database (graphic online at http://www.cdc.gov/ncidod/EID/vol7no3/sola_data.htm) summarizing the distribution of shared types (A), their respective sizes (B), and their relative distribution in different locations (C).

accounted for 4% of the isolates and were found in 11 countries. The remaining isolates (n=1,517) were scattered into 235 types. Figure 1B shows the relative sizes of 259 shared types; 109 shared types (42%) contained only two patients each and 38 shared types contained only three patients each. Inversely, 24 shared types containing >20 patients totaled 1,804 (65%) isolates. Finally, the distribution "unique" versus "ubiquitous" shared types (reported in one location versus found in two or more locations) is shown in Figure 1C; 122 (47%) shared types were reported from a single location, 69 (26%) were from two locations, and 25 (10%) were from three locations. Inversely, the most ubiquitous types, in increasing order of distribution, were 33 and 37, 20, 52, 42, 50, and 53. Thus, most *M. tuberculosis* shared types contained a low number of patient isolates and were confined geographically, whereas a minority contained a high number of patient isolates and were highly disseminated. The finding of identical spoligotypes in distant countries may be explained either by recent or past transmission events or by phylogenetic convergence. However, the evolution of the DR locus relies on at least three independent mechanisms, namely, homologous recombination (13), replication slippage (14, 15), and insertion sequence-mediated transposition (16-19), which does not favor a fortuitous convergence.

### Geographic Distribution of Shared Types in the Database

Analysis of geographic distribution of the shared types (see online graphic of database, http://www.cdc.gov/ncidod/EID/vol7no3/sola_data.htm) permitted us to split our collection into two broad categories: those reported in a single area (n=122, Table 2) and those reported in two or more areas (n=137). In the latter category, matching analysis for 69 spoligotypes found in four broad geographic areas, namely, Africa, the Americas (North, Central and Caribbean, and South America), Europe, and Asia (Middle East, and Far East Asia), is shown in Table 3. Contrary to ubiquitous spoligotypes such as type 1, 53, and 50, which have been found in all regions, this is an attempt to define potential inter-regional and inter-continental flow of *M. tuberculosis* isolates so far confined to limited geographic areas. The most frequent matches were found for clusters in European countries (n=17), followed by Europe and North America (n=8), Europe and Central America and the Caribbean (n=5), and Europe and South America (n=4) (Table 3). These matches may underline both recent transcontinental transmission events and the history of TB spread in the New World through European settlers.

A total of 25 shared types were reported in three countries. Among these, 8 types were exclusively found either in Europe (types 10,22,161) or the Americas (types 5,67,70,93,130); 10 types were shared between two European countries and a country of another region (types 35,49,59,86,115,118,136,138,139,150); 5 types were shared between two countries of the Americas with a country in Europe (types 92,119,168,185,190); 1 type was shared between a European country and two African countries (type 125); and 1 type was shared between Asia, Europe, and the USA (type 124). Finally, 15 types were found in four countries; 1 type (type 41) was exclusively found in Europe and may be specific for this continent. Fourteen other types were distributed as follows: Europe + Americas, 8 types (types 3,7,19,31,40,51,137,152); Europe + Africa, 1 type (type 21); Europe + Asia + Americas, 3 types (type 8,89,167); Europe + Americas + Africa, 1 type (type 64); and Europe + Africa + Asia, 1 type (type 126). Finally, 28 types were reported in five or more countries, suggesting that these types are widespread and may constitute the ubiquitous types such as the Beijing type (type 1 in our database) or the Haarlem type (type 47). The only exception in this category was type 17, which was found in six countries in the Americas and may be specific for this region. Future population studies should focus on these ubiquitous types to better define their relative prevalence in each country.

Table 2. Geographic distribution of potentially specific shared types of *Mycobacterium tuberculosis* reported in a single location (n=122)

| Region | Country | No. of types | Types |
|---|---|---|---|
| Americas | Guadeloupe | 7 | 12,13,14,15,30,103,259 |
| | French Guiana | 4 | 66,76,94,96 |
| | Cuba | 4 | 71,74,80,81 |
| | USA | 46 | 192,194,197-199,201,202, 205,206,208,210-217,219-235,237-239,241,243,246, 248,256-258 |
| Europe | The Netherlands | 4 | 9,18,28,90 |
| | United Kingdom | 6 | 16,23,27,38,43,100 |
| | France | 27 | 55,57,107-114,116,120,122, 140,141,143-148,170,171, 173,174,184,186 |
| | Italy | 9 | 155,157-160,163,165, 166,169 |
| | Spain | 2 | 104,106 |
| | Russia | 3 | 251,252,253 |
| Africa | Zimbabwe | 6 | 79,82-85,87 |
| | Guinea-Bissau | 1 | 188 |
| Asia | Philippines | 1 | 69 |
| | Mongolia | 2 | 97, 98 |

Table 3. Total number of matches found in matching analysis of the shared types (n=69) found at two geographic locations*

| | | Americas | | | | Asia* | |
|---|---|---|---|---|---|---|---|
| Matches* | Africa | North America | Central America/ Caribbean | South America | Europe | Middle East | Far East |
| Africa | 3[a] | 3[b] | 2[c] | 1[d] | 5[e] | 0 | 0 |
| North America | | NA+ | 6[f] | 4[g] | 8[h] | 0 | 1 |
| Central America | | | 2[i] | 4[j] | 5[k] | 0 | 0 |
| South America | | | | 3[l] | 4[m] | 0 | 0 |
| Europe | | | | | 17[n] | 1 | 0 |
| Asia (Middle East) | | | | | | 0 | 0 |
| Asia (Far East) | | | | | | | 0 |

*Indices a to n refer to the designation of the matching types. For full description of the matching shared type, see database (online graphic at http://www.cdc.gov/ncidod/EID/vol7no3/sola_data.htm). Spoligotyping data for isolates from Asia are scarce; hence, only two matches involving the Middle East and Far East were found (shared types 127 and 249, respectively). †NA, not applicable (matches were searched only for shared types existing between two countries or regions; as no data were available for Canada, comparison of isolates within North America was not feasible).

### Biogeographic Analysis of European Versus American Spoligotypes

Several possible scenarios could account for the introduction and spread of TB in the Americas; however, documented contact with Europeans is considered too recent to account for the widespread distribution of the disease by AD 1000 (20). One hypothesis suggests that TB may have penetrated the Americas through human migration from Asia via the Bering Strait (21). Another scenario suggests TB's initial introduction as a zoonosis that became an anthropozoonosis after cattle were domesticated (20,21). In this context, of the 259 shared types in our database, 59 were exclusively reported in the Americas, whereas 50 were found only in Europe (Table 2). This biogeographic dichotomy may signal the specific history of the disease in each continent. As enough data were present for the USA and Europe (2,418 [73%] isolates), a statistical analysis of distribution of shared types found in those two areas was performed.[1] Of 45 shared types in this category, results showed that differences in the distribution of certain shared types (1,19,20,25,26,37,44,48, 50,52,53,118,137) between the USA and Europe were highly significant, and sampling bias could not explain the differences observed (Table 4). On the other hand, the differences observed in the distribution of shared types 2,8, 33,34,47,58,62,92,138, and 139 between the USA and Europe were not statistically significant, and in this case sampling bias could not be fully excluded for the differences observed. Finally, our database described 58 isolates of the shared type 42 that were present in 11 countries (a ubiquitous type), but not a single isolate of type 42 was present among the 1,283 isolates from Texas (12).

Table 4. Analysis of distribution of shared types found in both USA and Europe[a]

| | Europe | | USA | | World | | |
|---|---|---|---|---|---|---|---|
| Type | No. (k1) | %[b] (p1) | No. (k2) | % (p2) | No. (p0) | % | $d/\sigma_{d\ quotient}$[c] |
| 1 | 21 | 1.8 | 326 | 25.5 | 476 | 14.4 | 15.3[d] |
| 2 | 6 | 0.5 | 2 | 0.2 | 28 | 0.8 | 1.6 |
| 8 | 10 | 0.9 | 7 | 0.5 | 19 | 0.6 | 0.9 |
| 19 | 1 | 0.1 | 23 | 1.8 | 27 | 0.8 | 4.2[d] |
| 20 | 8 | 0.7 | 2 | 0.2 | 20 | 0.6 | 2.1[d] |
| 25 | 13 | 1.1 | 3 | 0.2 | 17 | 0.5 | 2.7[d] |
| 26 | 22 | 1.9 | 5 | 0.4 | 28 | 0.8 | 3.6[d] |
| 33 | 13 | 1.1 | 10 | 0.8 | 38 | 1.2 | 0.9 |
| 34 | 6 | 0.5 | 9 | 0.7 | 21 | 0.6 | 0.6 |
| 37 | 17 | 1.5 | 2 | 0.2 | 28 | 0.8 | 3.7[d] |
| 44 | 12 | 1.1 | 1 | 0.1 | 15 | 0.5 | 3.3[d] |
| 47 | 25 | 2.2 | 23 | 1.8 | 65 | 2.0 | 0.7 |
| 48 | 34 | 3.0 | 7 | 0.5 | 41 | 1.2 | 4.6[d] |
| 50 | 56 | 4.9 | 32 | 2.5 | 155 | 4.7 | 3.1[d] |
| 52 | 29 | 2.5 | 7 | 0.5 | 40 | 1.2 | 4.0[d] |
| 53 | 79 | 6.9 | 46 | 3.6 | 218 | 6.6 | 3.6[d] |
| 58 | 4 | 0.4 | 7 | 0.5 | 17 | 0.5 | 0.7 |
| 62 | 7 | 0.6 | 4 | 0.3 | 15 | 0.5 | 1.1 |
| 92 | 2 | 0.2 | 8 | 0.6 | 14 | 0.4 | 1.7 |
| 118 | 8 | 0.7 | 1 | 0.1 | 9 | 0.3 | 2.5[d] |
| 119 | 2 | 0.2 | 110 | 8.6 | 115 | 3.5 | 9.6[d] |
| 137 | 10 | 0.9 | 134 | 10.5 | 146 | 4.4 | 9.7[d] |
| 138 | 5 | 1 | 1 | 0.1 | 6 | 0.2 | 1.8 |
| 139 | 19 | 1.7 | 19 | 1.5 | 38 | 1.2 | 0.3 |

[a]Results are given for 24 of 45 shared types that contained enough isolates to compare the results statistically.
[b]Percentages were calculated on the basis of 1,142 (n1), 1,276 (n2), and 3,319 individual spoligotypes reported, respectively, for Europe (p1), USA (p2), and the full database available for the world.
[c]The quotient $d/\sigma_d$ was calculated using the equation $d/\sigma_d=p_1-p_2/\sqrt{p_0q_0(1/n_1+1/n_2)}$, where d is the absolute value of the difference between p1 and p2, $\sigma_d$ is the standard deviation of the repartition law of d which follows a normal distribution and can be calculated by the equation $\sigma_d=\sqrt{p_0q_0\,(1/n_1+1/n_2)}$, and where $p_0$ is best estimated by the equation $p_0=k_1+k_2/n_1+n_2=n_1p_1+n_2p_2/n_1+n_2$. In this equation, individual sampling sizes are $n_1$ and $n_2$, the number of individuals within a given shared-type "x" are $k_1$ and $k_2$, and the representativeness for the two samples is $p_1=k_1/n_1$ and $p_2=k_2/n_2$.
[d]If the absolute value of the quotient $d/\sigma_d<2$, the variations observed in the distribution of isolates for a given shared type were not statistically significant and could be due to a sampling bias. Inversely, if $d/\sigma_d>2$, then the differences observed in the distribution of isolates for a given shared type were statistically significant and not due to a potential sample bias.

### Use of Database for Epidemiologic Studies

Essentially working in a Caribbean setting for last 6 years with systematic typing of all *M. tuberculosis* isolates from Guadeloupe, Martinique, and French Guiana, we initially focused on spoligotypes that may be specific to our region. Of 259 shared types, 85 types were present in the Caribbean. Of these, 69 were common to the Caribbean and the rest of the world, and 16 were reported only from the Caribbean (types 5,12,13,14,15,30,63,66,68,72,76,77,94, 96,103,259). Although TB has a penchant to be latent for years or decades, because of an exhaustive (nearly 100%) recruitment of isolates from the French Caribbean for last 6 years, finding a previously unreported spoligotype in our region may constitute indirect evidence for a newly imported case of TB in most instances, particularly if an epidemiologic investigation does not suggest reactivation of old disease.

As far as global epidemiologic studies are concerned, this database also emphasizes the existence of highly prevalent families of *M. tuberculosis* isolates, e.g., the Beijing type, which represents a diverse collection of clones including the notorious multidrug-resistant strain W and other W-like drug-sensitive isolates (5,22). Studies focusing on *M. tuberculosis* isolates from developing countries, where TB is highly prevalent, would improve understanding of the worldwide circulation of tubercle bacilli and provide insights into their epidemiology, phylogeny, and virulence.

### Phylogenetic Reconstruction of *M. tuberculosis*

For phylogenetic analysis (23), a neighbor-joining tree was constructed by calculating the 1-Jaccard Index (10,24). This tree (Figure 2) incorporates the data for 252 *M. tuberculosis* shared types instead of the 259 allele types

[1]For this purpose, the independent sampling sizes for Europe and the USA were taken as $n_1$ and $n_2$, the number of individuals within a given shared-type "x" was $k_1$ and $k_2$, and in this case, the representativeness of the two samples was $p_1=k_1/n_1$ and $p_2=k_2/n_2$, respectively. To assess if the divergence observed between $p_1$ and $p_2$ was due to sampling bias or the existence of two distinct populations, the percentage of individuals ($p_0$) harboring shared-type "x" in the population studied was estimated by the equation $p_0= k_1+k_2/n_1+n_2=n_1p_1+n_2p_2/n_1+n_2$. The distribution of the percentage of shared-type "x" in the sample sizes $n_1$ and $n_2$ follows a normal distribution with a mean $p_0$ and a standard deviation of $\sqrt{p_0q_0/n_1}$ and $\sqrt{p_0q_0/n_2}$, respectively, and the difference $d=p_1-p_2$ follows a normal distribution of mean $p_0-p_0=0$ and of variance $\sigma_d^2=\sigma_{p1}^2+\sigma_{p2}^2=p_0q_0/n_1+p_0q_0/n_2$ or $\sigma_d^2=p_0q_0\,(1/n_1+1/n_2)$. The two samples being independent, the two variances were additive; the standard deviation $\sigma d=\sqrt{p_0q_0\,(1/n_1+1/n_2)}$ was calculated, and the homogeneity of the samples tested was assessed using the quotient $d/\sigma_d=p_1-p_2/\sqrt{p_0q_0(1/n_1=1/n_2)}$. If the absolute value of the quotient $d/\sigma_d<2$, the two samples were considered to belong to the same population (CI 95%) and the variation observed in the distribution of isolates for given shared types could be due to a sampling bias. Inversely, if $d/\sigma_d>2$, then the differences observed in the distribution of isolates for given shared types were statistically significant and not due to potential sample bias.

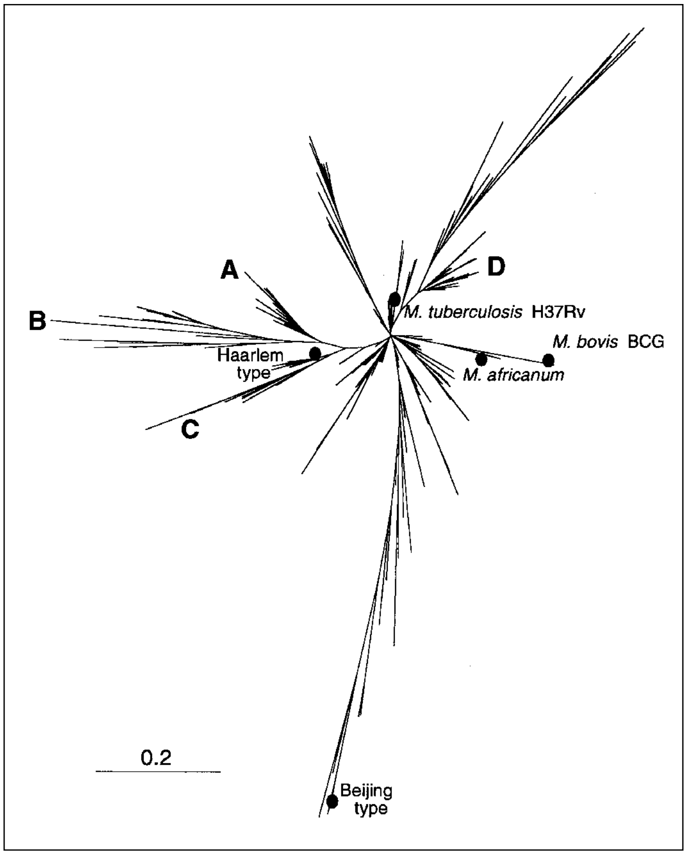

Figure 2. Phylogenetic tree of shared types of *Mycobacterium tuberculosis* constructed by pairwise comparison of patterns using the "1-Jaccard" index and the neighbor-joining algorithm. Approximately 15 branches may be visualized at an arbitrary distance of 0.2. The position of some reference strains (*M. tuberculosis* H37Rv, *M. bovis* BCG) or well-studied spoligotyping families of isolates (Beijing, Haarlem, and the *M. africanum* group) are also indicated.

described in the online database (types 253 to 259 were added recently after the completion of phylogenetic analysis). At an arbitrary distance of 0.2, one may easily distinguish nearly 15 branches that may contain significant phylogenetic information, as seen below for four selected branches (A to D) by combining results using independent genetic markers (Figure 3). As shown in Figure 2 and 3A, the homogeneous branch A (mainly present in Europe, West Africa, and South America) contains 20 types characterized by the absence of spacers 29 to 32 and 34. Such a family of isolates was recently described in Guinea-Bissau and also found to harbor a low copy number of IS*6110* (25). Information concerning *katG*283-*gyrA*95 allele combination was available for 5 of these 20 types and showed that branch A belonged to the major genotypic group 1 as defined previously (26) and may represent an ancestral clone of *M. tuberculosis* isolates originating in Africa, Asia, or both (27; this work). For this branch, VNTR information was available for 3 of 20 types and showed a high exact tandem repeat (ETR)-A copy number (between 4 to 7; Figure 3A), which is common both for *M. bovis* and *M. africanum* (8,28).

Branch B shared a common root with branch A (Figure 2) but was clearly distinct from the population in branch A, an observation corroborated both by VNTR and *katG*283-*gyrA*95 types (Figure 3B). All the isolates in branches A and B were of the major genetic group 1, as defined (26), except for a single isolate of the major genetic group 2 in branch B (type 199); the significance of this observation is not clear. Branch C was composed of two subbranches, which are likely to be of different phylogenetic significance (Figure 3C); the upper part related to the Haarlem family, as previously defined (15), and was highly homogeneous upon VNTR typing (alleles 32333), whereas the lower part was quite heterogenous (alleles 42431, 31333, 44553).

Finally, branch D comprised a subfamily of the spoligotypes that all missed spacers 33-36 (Figure 3D). This branch, which contained 30 different shared types, was easily

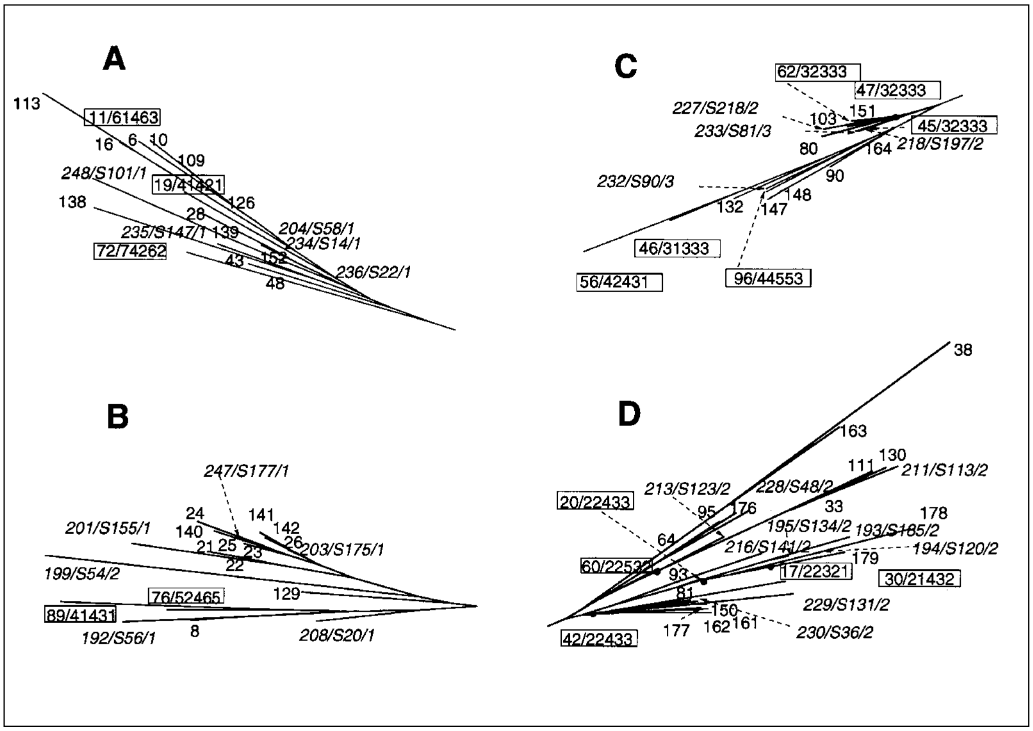

Figure 3. Enlargement of branches A to D from the *Mycobacterium tuberculosis* phylogenetic tree (Figure 2). Numbers in standard characters refer to spoligotype numbers according to our database; those in boxes describe both the spoligotype number and variable number of tandem DNA repeats (VNTR) allele designations. Italicized numbers refer to spoligotype followed by the Houston spoligotype designation (12), and the major genetic groups 1 to 3 defined on the basis of *katG*283-*gyrA*95 allele combination (24). A and B show distinct branches belonging essentially to the major genetic group 1 with a high exact tandem repeat (ETR)-A copy number; C and D show branches that include some strains of the "Haarlem family" belonging to the major genetic group 2 with a low ETR-A copy number.

characterized by simultaneous absence of spacers 21-24 and 33-36, and constitutes a highly ramified but homogeneous family on the basis of its belonging to the major genetic group 2 of Sreevatsan et al. (26), and the presence of two copies of the ETR-A allele upon VNTR typing. Frequently found in southern Europe and Central and South America, the ancestral type of this family (type 42) may have evolved by stepwise mutation to give, successively, types 20 and 17 (Figure 3D). This assumption is corroborated by the position of the respective types in the tree and their spoligotyping and VNTR patterns; type 42 (all spacers present except 21 to 24 and 33 to 36, VNTR 22433), type 20 (identical to type 42 plus a single missing spacer 3, VNTR identical to type 42), and type 17 (identical to type 20 plus a single missing spacer 13, VNTR 22321).

These results show that branches A and B are likely to be of an older evolutionary origin than branches C and D. Källenius et al. (25) hypothesized that branches A and B could find their evolutionary origin in West Africa, whereas branches C and D could be of European descent. However, since the global evolutionary rate of the DR locus may involve many independent mechanisms, this tree is likely to incorporate systematic yet unknown errors (6); therefore, a detailed analysis of the robustness of each potential phylogenetic link is under investigation.

### Conclusion

We have presented an update of a database of *M. tuberculosis* spoligotypes with a detailed description of 259 shared types. This database may help to address major aspects linked to recent mycobacterial reemergence, evolutionary history, and future epidemiologic studies. Our results demonstrate that a few major families of conserved spoligotypes are well distributed throughout the world, whereas others are specific for certain geographic regions. Thus, the current epidemiologic picture of TB appears to be based both on the persistence of ancestral clones of *M. tuberculosis* as well as those emerging more recently, e.g., the Beijing type (type 1 in our database), which also includes the MDR strain W from New York City. A future correlation between genotyping and resistance data and the respective prevalence of various clones region by region may provide more insight into the global circulation of TB and help establish priorities in TB control programs. For example, because we have typed all *M. tuberculosis* clinical isolates in our insular setting for last 6 years, introduction of a previously unreported clone in Guadeloupe may be detected and, when placed in epidemiologic context, may either be classified as a newly imported case of TB or as a reactivation. Simultaneously, an epidemiologic investigation around the case is immediately initiated by local health authorities. A comparison of the newly imported clone with those in the database sometimes suggests a probable link to a specific community or, alternatively, regional, national, or intercontinental importation of the disease.

Concerning the global phylogeny of *M. tuberculosis*, the pairwise comparison of the 252 shared types by calculation of the 1-Jaccard index and the neighbor-joining algorithm underscored phylogenetic relationships between some of the families of spoligotypes described. Four major families of spoligotypes (branches A-D) were discussed in detail, and the results were corroborated by VNTR and *katG—gyrA* polymorphism data, which support the robustness of the branchings proposed. Nevertheless, a detailed and more exhaustive analysis of evolutionary and historical spreading of the different families of tubercle bacilli is a long-term goal requiring a never-ending compilation of data. Ideally, this database could be expanded to incorporate detailed *M. bovis* and *M. africanum* results so as to infer the global phylogeny of all members of the *M. tuberculosis* complex.

It has been suggested that the evolutionary rate of *M. tuberculosis* may be strain dependent (29). In this context, our investigation also pointed out a previously unnoticed link between spoligotypes and the *katG—gyrA* polymorphism (Figure 3), i.e., the isolates in the spoligotyping-defined branch A belonged to the major genetic group 1 of Sreevatsan et al. (26), whereas those in branch D belonged to the major genetic group 2. Since the isolates in these branches came from diverse geographic areas, we suggest that the pace of the molecular clock of the DR locus might be much slower than that of other markers, such as IS*6110*. This assumption is supported by a recent study on the evolutionary origin of the DR locus of *M. tuberculosis* (19). Finally, by comparing observations with outcomes of a stepwise mutation model, the insertion sequences of the tubercle bacilli are far from equilibrium; indeed, transposition parameters appear to have a much stronger effect on IS*6110* copy number distribution than epidemic parameters and have a direct action on bacterial diversity of the *M. tuberculosis* complex (30). New studies are needed to clarify the complex relationships between epidemic parameters, selection factors, and genomic evolutionary mechanisms of the tubercle bacilli.

### Acknowledgments

We thank Olga Narvskaya, Sofia Samper, Carlos Martin, Bernard Carbonnelle, and Jérôme Maïsetti for permission to use their unpublished results in our database and to Prasitt Palittapongarnpim and Richard Frothingham for providing some of the DNAs used for spoligotyping and permission to use their unpublished results in our database.

This work was supported through grants by the Délégation Générale au Réseau International des Instituts Pasteur et Instituts Associés, Institut Pasteur, Paris, and Fondation Française Raoul Follereau, Paris, France.

Dr. Sola is a senior scientist at the Pasteur Institute and has been working at the Institut Pasteur de Guadeloupe for the last 6 years. His current research interest focuses on molecular population genetics of tubercle bacilli for public health and academic purposes.

### References

1. World Health Organization. Global tuberculosis control. WHO report 1999. Geneva: The Organization; 1999.
2. Slutkin G. Global AIDS 1981-1999: the response. Int J Tuberc Lung Dis 2000;4:S24-S33.
3. Snider DE, Castro KG. The global threat of drug resistant tuberculosis. N Engl J Med 1998;338:1689-90.
4. Long R, Nobert E, Chomyc S, van Embden J, McNamee C, Duran RR, et al. Transcontinental spread of multidrug-resistant *Mycobacterium bovis.* Am J Respir Crit Care Med 1999;159:2014-17.
5. Bifani PJ, Mathema B, Liu Z, Moghazeh SL, Shopsin B, Tempalski B, et al. Identification of a W variant outbreak of *Mycobacterium tuberculosis* via population-based molecular epidemiology. JAMA 1999;282:2321-7.

6. Sola C, Devallois A, Horgen L, Maïsetti J, Filliol I, Legrand E, et al. Tuberculosis in the Carribean: using spacer oligonucleotide typing to understand strain origin and transmission. Emerg Infect Dis 1999;5:404-14.
7. Kamerbeek J, Schouls L, Kolk A, van Agterveld M, van Soolingen D, Kuijper S, et al. Simultaneous detection and strain differentiation of *Mycobacterium tuberculosis* for diagnosis and epidemiology. J Clin Microbiol 1997;35:907-14.
8. Frothingham R, Meeker-O'Connell WA. Genetic diversity in the *Mycobacterium tuberculosis* complex based on variable numbers of tandem DNA repeats. Microbiology 1998;144:1189-96.
9. Grimont PAD. TAXOTRON instruction manual. Paris: Taxolab, Institut Pasteur; 1996.
10. Jaccard P. Nouvelles recherches sur la distribution florale. Bulletin de la Société Vaudoise de Sciences Naturelles 1908;44:223-70.
11. Saitou N, Nei M. The neighbor-joining method: a new method for reconstructing phylogenetic trees. Mol Biol Evol 1987;4:406-25.
12. Soini H, Pan X, Amin A, Graviss EA, Siddiqui A, Musser JM. Characterization of *Mycobacterium tuberculosis* isolates from patients in Houston, Texas, by spoligotyping. J Clin Microbiol 2000;38:669-76.
13. Groenen PMA, Bunschoten AE, van Soolingen D, van Embden JDA. Nature of DNA polymorphism in the direct repeat cluster of *Mycobacterium tuberculosis*; application for strain differentiation by a novel typing method. Mol Microbiol 1993;10:1057-65.
14. Hancock JM. The contribution of slippage-like processes to genome evolution. J Mol Evol 1995;41:1038-47.
15. Kremer K, van Soolingen D, Frothingham R, Haas WH, Hermans PWM, Martin C, et al. Comparison of methods based on different molecular epidemiological markers for typing of *Mycobacterium tuberculosis* strains: interlaboratory study of discriminatory power and reproducibility. J Clin Microbiol 1999;37:2607-18.
16. Fang Z, Doig C, Kenna DT, Smittipat N, Palittapongarnpim P, Watt B, et al. IS*6110*-mediated deletions of wild-type chromosomes of *Mycobacterium tuberculosis.* J Bacteriol 1999;181:1014-20.
17. Fang Z, Morrison N, Watt B, Doig C, Forbes KJ. IS*6110* transposition and evolutionary scenario of the direct repeat locus in a group of closely related *Mycobacterium tuberculosis* strains. J Bacteriol 1998;180:2102-9.
18. Filliol I, Sola C, Rastogi N. Detection of a previously unamplified spacer within the DR locus of *Mycobacterium tuberculosis*: Epidemiological implications. J Clin Microbiol 2000;38:1231-4.
19. van Embden JDA, van Gorkom T, Kremer K, Jansen R, Van der Zeijst BAM, Schouls LM. Genetic variation and evolutionary origin of the direct repeat locus of *Mycobacterium tuberculosis* complex bacteria. J Bacteriol 2000;182:2393-01.
20. Buikstra JE. Paleoepidemiology of tuberculosis in the Americas. In: Palfi G, Dutour O, Deak J, Hutas I, editors. Tuberculosis: past and present. Szeged, Hungary: Golden Book Publisher Ltd.; 1999. p. 479-94.
21. Ortner DJ. Paleopathology: implications for the history and evolution of tuberculosis. In: Palfi G, Dutour O, Deak J, Hutas I, editors. Tuberculosis: past and present. Szeged, Hungary: Golden Book Publisher Ltd; 1999. p. 255-61.
22. Kurepina NE, Sreevatsan S, Plikaytis BB, Bifani PB, Connell ND, Donneelly RJ, et al. Characterization of the phylogenetic distribution and chromosomal insertion sites of five IS*6110* elements in *Mycobacterium tuberculosis*: non random integration in the *dna*A-*dna*N region. Tuber Lung Dis 1998;79:31-42.
23. Nei M. Phylogenetic analysis in molecular evolutionary genetics. Annu Rev Genet 1996;30:371-403.
24. Saitou N, Nei M. The neighbor-joining method: a new method for reconstructing phylogenetic trees. Mol Biol Evol 1987;4:406-25.
25. Källenius G, Koivula T, Ghebremichael S, Hoffner SE, Norberg R, Svensson E, et al. Evolution and clonal traits of *Mycobacterium tuberculosis* in Guinea-Bissau. J Clin Microbiol 1999;37:3872-8.
26. Sreevatsan S, Pan X, Stockbauer K, Connell N, Kreiswirth B, Whittam T, et al. Restricted structural gene polymorphism in the *Mycobacterium tuberculosis* complex indicates evolutionarily recent global dissemination. Proc Natl Acad Sci U S A 1997;97:9869-74.
27. Castets M, Boisvert H, Grumbach F, Brunel M, Rist N. Les bacilles tuberculeux de type africain: note préliminaire. Revue de la Tuberculose et de Pneumologie 1968;32:179-84.
28. Frothingham R, Strickland PL, Bretzel G, Ramaswamy S, Musser JM, Williams DL. Phenotypic and genotypic characterization of *Mycobacterium africanum* isolates from West Africa. J Clin Microbiol 1999;37:1921-6.
29. Warren GM, Richardson M, Sampson S, Bourn W, van der Spuy G, Hide W, et al. RFLP analysis of *M. tuberculosis* demonstrates strain-dependent evolution. Int J Tuberc Lung Dis 1999;3(Suppl. I);S38.
30. Tanaka MM, Small PM, Salamon H, Feldman MW. The dynamics of repeated elements: applications to the epidemiology of tuberculosis. Proc Natl Acad Sci U S A 2000;97:3532-7.
31. Bauer J, Andersen AB, Kremer K, Miörner H. Usefulness of spoligotyping to discriminate IS*6110* low-copy-number *Mycobacterium tuberculosis* complex strains cultures in Denmark. J Clin Microbiol 1999;37:2602-6.
32. Bonora S, Gutierrez MC, Perri GD, Brunello F, Allegranzi B, Ligozzi M, et al. Comparative evaluation of Ligation-mediated PCR and spoligotyping as screening methods for genotyping of *Mycobacterium tuberculosis* strains. J Clin Microbiol 1999;37:3118-23.
33. Diaz R, Kremer K, de Haas PEW, Gomez RI, Marrero A, Valdivia JA, et al. Molecular epidemiology of tuberculosis in Cuba outside of Havana, July 1994-June 1995: utility of spoligotyping versus IS*6110* restriction fragment length polymorphism. Int J Tuberc Lung Dis 1998;2:743-50.
34. Douglas JT, Qian L, Montoya JC, Sreevatsan S, Musser J, van Soolingen D, et al. Detection of a novel family of tuberculosis isolates in the Philippines. 97th general meeting of the American Society for Microbiology. Washington: ASM Press; 1997. p.572.
35. Escalante P, Ramaswamy S, Sanabria H, Soini H, Pan X, Valiente-Castillo O, et al. Genotypic characterization of drug-resistant *Mycobacterium tuberculosis* isolates from Peru. Tuber Lung Dis 1998;79:111-18.
36. Goguet dela Salmonière YO, Li HM, Torrea G, Bunschoten A, van Embden JDA, Gicquel B. Evaluation of spoligotyping in a study of the transmission of *Mycobacterium tuberculosis.* J Clin Microbiol 1997;35:2210-14.
37. Goyal M, Saunders NA, van Embden JDA, Young DB, Shaw RJ. Differentiation of *Mycobacterium tuberculosis* isolates by spoligotyping and IS*6110* restriction fragment length polymorphism. J Clin Microbiol 1997;35:647-51.
38. Heyderman RS, Goyal M, Roberts P, Ushewokunze S, Zishou S, Marshall BG, et al. Pulmonary tuberculosis in Harare, Zimbabwe: analysis by spoligotyping. Thorax 1998;53:346-50.
39. Niang MN, Goguet de la Salmonière YO, Samb A, Hane AA, Cisse MF, Gicquel B, et al. Characterization of *M. tuberculosis* strains from West-African patients by spoligotyping. Microbes Infect 1999;1:1189-92.
40. Popa MI, Goguet de la Salmonière YO, Teodor I, Popa L, Stefan M, Banica D, et al. Genomic profile of Romanian *M. tuberculosis* strains appreciated by spoligotyping. Rom Arch Microbiol Immunol 1997;56:63-75.
41. de C Ramos M, Soini H, Roscanni GC, Jaques M, Villares MC, Musser JM. Extensive cross-contamination of specimens with *Mycobacterium tuberculosis* in a reference laboratory. J Clin Microbiol 1999;37:916-19.
42. Taylor GM, Goyal M, Legge AJ, Shaw RJ, Young D. Genotypic analysis of *Mycobacterium tuberculosis* from medieval human remains. Microbiology 1999;145:899-904.
43. van der Zanden AG, Hoentgen AH, Heilmann FG, Weltvreden EF, Schouls LM, van Embden JD. Simultaneous detection and strain differentiation of *Mycobacterium tuberculosis* complex in paraffin wax embedded tissues and in stained microscopic preparations. Mol Pathol 1998;51:209-14.
44. van Soolingen D, Qian L, de Haas PEW, Douglas JT, Traore H, Portaels F, et al. Predominance of a single genotype of *Mycobacterium tuberculosis* in countries of East Asia. J Clin Microbiol 1995;33;3234-8.

## References

[PDF_00727] Bauer J., Andersen A. B., Kremer K., Miörner H. (1999). Usefulness of spoligotyping To discriminate IS6110 low-copy-number Mycobacterium tuberculosis complex strains cultured in Denmark.. J Clin Microbiol.

[PDF_00638] Bifani P. J., Mathema B., Liu Z., Moghazeh S. L., Shopsin B., Tempalski B., Driscol J., Frothingham R., Musser J. M., Alcabes P. (1999). Identification of a W variant outbreak of Mycobacterium tuberculosis via population-based molecular epidemiology.. JAMA.

[PDF_00731] Bonora S., Gutierrez M. C., Di Perri G., Brunello F., Allegranzi B., Ligozzi M., Fontana R., Concia E., Vincent V. (1999). Comparative evaluation of ligation-mediated PCR and spoligotyping as screening methods for genotyping of Mycobacterium tuberculosis strains.. J Clin Microbiol.

[PDF_00714] Castets M., Boisvert H., Grumbach F., Brunel M., Rist N. (1968). Les bacilles tuberculeux de type Africain: note préliminaire.. Rev Tuberc Pneumol (Paris).

[PDF_00735] Diaz R., Kremer K., de Haas P. E., Gomez R. I., Marrero A., Valdivia J. A., van Embden J. D., van Soolingen D. (1998). Molecular epidemiology of tuberculosis in Cuba outside of Havana, July 1994-June 1995: utility of spoligotyping versus IS6110 restriction fragment length polymorphism.. Int J Tuberc Lung Dis.

[PDF_00744] Escalante P., Ramaswamy S., Sanabria H., Soini H., Pan X., Valiente-Castillo O., Musser J. M. (1998). Genotypic characterization of drug-resistant Mycobacterium tuberculosis isolates from Peru.. Tuber Lung Dis.

[PDF_00676] Fang Z., Doig C., Kenna D. T., Smittipat N., Palittapongarnpim P., Watt B., Forbes K. J. (1999). IS6110-mediated deletions of wild-type chromosomes of Mycobacterium tuberculosis.. J Bacteriol.

[PDF_00679] Fang Z., Morrison N., Watt B., Doig C., Forbes K. J. (1998). IS6110 transposition and evolutionary scenario of the direct repeat locus in a group of closely related Mycobacterium tuberculosis strains.. J Bacteriol.

[PDF_00683] Filliol I., Sola C., Rastogi N. (2000). Detection of a previously unamplified spacer within the DR locus of Mycobacterium tuberculosis: epidemiological implications.. J Clin Microbiol.

[PDF_00652] Frothingham R., Meeker-O'Connell W. A. (1998). Genetic diversity in the Mycobacterium tuberculosis complex based on variable numbers of tandem DNA repeats.. Microbiology.

[PDF_00717] Frothingham R., Strickland P. L., Bretzel G., Ramaswamy S., Musser J. M., Williams D. L. (1999). Phenotypic and genotypic characterization of Mycobacterium africanum isolates from West Africa.. J Clin Microbiol.

[PDF_00748] Goguet de la Salmonière Y. O., Li H. M., Torrea G., Bunschoten A., van Embden J., Gicquel B. (1997). Evaluation of spoligotyping in a study of the transmission of Mycobacterium tuberculosis.. J Clin Microbiol.

[PDF_00752] Goyal M., Saunders N. A., van Embden J. D., Young D. B., Shaw R. J. (1997). Differentiation of Mycobacterium tuberculosis isolates by spoligotyping and IS6110 restriction fragment length polymorphism.. J Clin Microbiol.

[PDF_00665] Groenen P. M., Bunschoten A. E., van Soolingen D., van Embden J. D. (1993). Nature of DNA polymorphism in the direct repeat cluster of Mycobacterium tuberculosis; application for strain differentiation by a novel typing method.. Mol Microbiol.

[PDF_00669] Hancock J. M. (1995). The contribution of slippage-like processes to genome evolution.. J Mol Evol.

[PDF_00756] Heyderman R. S., Goyal M., Roberts P., Ushewokunze S., Zizhou S., Marshall B. G., Makombe R., Van Embden J. D., Mason P. R., Shaw R. J. (1998). Pulmonary tuberculosis in Harare, Zimbabwe: analysis by spoligotyping.. Thorax.

[PDF_00648] Kamerbeek J., Schouls L., Kolk A., van Agterveld M., van Soolingen D., Kuijper S., Bunschoten A., Molhuizen H., Shaw R., Goyal M. (1997). Simultaneous detection and strain differentiation of Mycobacterium tuberculosis for diagnosis and epidemiology.. J Clin Microbiol.

[PDF_00671] Kremer K., van Soolingen D., Frothingham R., Haas W. H., Hermans P. W., Martín C., Palittapongarnpim P., Plikaytis B. B., Riley L. W., Yakrus M. A. (1999). Comparison of methods based on different molecular epidemiological markers for typing of Mycobacterium tuberculosis complex strains: interlaboratory study of discriminatory power and reproducibility.. J Clin Microbiol.

[PDF_00698] Kurepina N. E., Sreevatsan S., Plikaytis B. B., Bifani P. J., Connell N. D., Donnelly R. J., van Sooligen D., Musser J. M., Kreiswirth B. N. (1998). Characterization of the phylogenetic distribution and chromosomal insertion sites of five IS6110 elements in Mycobacterium tuberculosis: non-random integration in the dnaA-dnaN region.. Tuber Lung Dis.

[PDF_00707] Källenius G., Koivula T., Ghebremichael S., Hoffner S. E., Norberg R., Svensson E., Dias F., Marklund B. I., Svenson S. B. (1999). Evolution and clonal traits of Mycobacterium tuberculosis complex in Guinea-Bissau.. J Clin Microbiol.

[PDF_00635] Long R., Nobert E., Chomyc S., van Embden J., McNamee C., Duran R. R., Talbot J., Fanning A. (1999). Transcontinental spread of multidrug-resistant Mycobacterium bovis.. Am J Respir Crit Care Med.

[PDF_00703] Nei M. (1996). Phylogenetic analysis in molecular evolutionary genetics.. Annu Rev Genet.

[PDF_00759] Niang M. N., de la Salmoniere Y. G., Samb A., Hane A. A., Cisse M. F., Gicquel B., Perraut R. (1999). Characterization of M. tuberculosis strains from west African patients by spoligotyping.. Microbes Infect.

[PDF_00763] Popa M. I., Goguet Y., Teodor I., Popa L., Stefan M., Bãnicã D., Gicquel B. (1997). Genomic profile of Romanian M. tuberculosis strains appreciated by spoligotyping.. Roum Arch Microbiol Immunol.

[PDF_00659] Saitou N., Nei M. (1987). The neighbor-joining method: a new method for reconstructing phylogenetic trees.. Mol Biol Evol.

[PDF_00705] Saitou N., Nei M. (1987). The neighbor-joining method: a new method for reconstructing phylogenetic trees.. Mol Biol Evol.

[PDF_00631] Slutkin G. (2000). Global AIDS 1981-1999: the response.. Int J Tuberc Lung Dis.

[PDF_00633] Snider D. E., Castro K. G. (1998). The global threat of drug-resistant tuberculosis.. N Engl J Med.

[PDF_00661] Soini H., Pan X., Amin A., Graviss E. A., Siddiqui A., Musser J. M. (2000). Characterization of Mycobacterium tuberculosis isolates from patients in Houston, Texas, by spoligotyping.. J Clin Microbiol.

[PDF_00644] Sola C., Devallois A., Horgen L., Maïsetti J., Filliol I., Legrand E., Rastogi N. (1999). Tuberculosis in the Caribbean: using spacer oligonucleotide typing to understand strain origin and transmission.. Emerg Infect Dis.

[PDF_00710] Sreevatsan S., Pan X., Stockbauer K. E., Connell N. D., Kreiswirth B. N., Whittam T. S., Musser J. M. (1997). Restricted structural gene polymorphism in the Mycobacterium tuberculosis complex indicates evolutionarily recent global dissemination.. Proc Natl Acad Sci U S A.

[PDF_00724] Tanaka M. M., Small P. M., Salamon H., Feldman M. W. (2000). The dynamics of repeated elements: applications to the epidemiology of tuberculosis.. Proc Natl Acad Sci U S A.

[PDF_00771] Taylor G. M., Goyal M., Legge A. J., Shaw R. J., Young D. (1999). Genotypic analysis of Mycobacterium tuberculosis from medieval human remains.. Microbiology.

[PDF_00767] de C Ramos M., Soini H., Roscanni G. C., Jaques M., Villares M. C., Musser J. M. (1999). Extensive cross-contamination of specimens with Mycobacterium tuberculosis in a reference laboratory.. J Clin Microbiol.

[PDF_00686] van Embden J. D., van Gorkom T., Kremer K., Jansen R., van Der Zeijst B. A., Schouls L. M. (2000). Genetic variation and evolutionary origin of the direct repeat locus of Mycobacterium tuberculosis complex bacteria.. J Bacteriol.

[PDF_00779] van Soolingen D., Qian L., de Haas P. E., Douglas J. T., Traore H., Portaels F., Qing H. Z., Enkhsaikan D., Nymadawa P., van Embden J. D. (1995). Predominance of a single genotype of Mycobacterium tuberculosis in countries of east Asia.. J Clin Microbiol.

[PDF_00774] van der Zanden A. G., Hoentjen A. H., Heilmann F. G., Weltevreden E. F., Schouls L. M., van Embden J. D. (1998). Simultaneous detection and strain differentiation of Mycobacterium tuberculosis complex in paraffin wax embedded tissues and in stained microscopic preparations.. Mol Pathol.

